# Unveiling the mechanisms of ultrasonic radiation-induced free radical stress on algal communities: Insights into growth inhibition, photosynthetic disruption, and antioxidant defense responses

**DOI:** 10.1016/j.ultsonch.2025.107297

**Published:** 2025-03-01

**Authors:** Xiaoge Wu, Tingting Shen, Xiaoyang Liu, Guangming Zhang, Xiaoqing Qian, Wenlan Yang

**Affiliations:** a College of Environmental Science and Engineering, Yangzhou University, Yangzhou 225009, China; b School of Energy & Environmental Engineering, Hebei University of Technology, Tianjin 300130, China; c Key Laboratory of Cultivated Land Quality Monitoring and Evaluation, Ministry of Agriculture and Rural Affairs, Yangzhou 225009, China

**Keywords:** Ultrasonic radiation, Mixed algal cultures, Free Radical, Antioxidant responses, Genomic analysis

## Abstract

Algal blooms pose a significant threat to global environmental health, compromising water quality and public safety. Ultrasonic radiation has emerged as a promising, eco-friendly strategy for controlling these blooms, but the underlying mechanisms remain unclearly understood. This study investigated the effects of ultrasonic radiation on the growth, photosynthetic performance, and antioxidant defense systems of an algal mixture over a 5-day period. Analysis techniques, including scanning electron microscopy (SEM), excitation-emission matrix (EEM) analysis, and transcriptomic profiling, were employed to elucidate the multifaceted responses of algal cells to ultrasonic treatment. Ultrasonic radiation induced significant free radical generation, primarily hydroxyl radicals (·OH), which played a critical role in cellular damage. Within 24 h, treatment led to a 50% reduction in algal cell counts, a 30% decline in chlorophyll-a levels, and a 25% decrease in photosynthetic efficiency. Phycocyanin, a vital pigment for cyanobacteria, exhibited heightened sensitivity to a single ultrasonic treatment, while subsequent treatments showed no additional reduction, suggesting that *Microcystis aeruginosa* is particularly susceptible to the ultrasonic damage. EEM analysis revealed significant changes in the fluorescence intensity of extracellular organic matter (EOM) and intracellular organic matter (IOM) peaks, indicative of oxidative stress and metabolic disruption. Transcriptomic analysis of *Microcystis aeruginosa* revealed a profound reprogramming of gene expression in response to sonication. Stress response genes, particularly those involved in antioxidant defense, were upregulated, while photosynthesis-related genes were downregulated. Our research indicates that short-term ultrasonic radiation has a long-term stress effect on algal cells, and this might be able to prevent the tendency of cyanobacteria blooms.

## Graphical abstract

**Figure:**
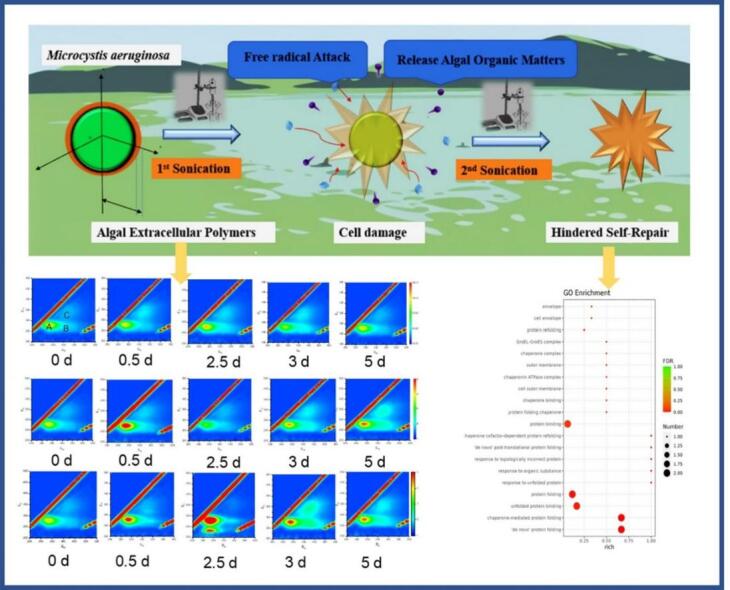

## 1 Introduction

The issue of algal blooms in water bodies worldwide is exceptionally severe [12], [21], [29]. The excessive growth of algae not only harm aquatic ecosystems but also poses threats to water supply and water safety. In response to algae, common treatment technologies are divided into two application scenarios: one is to directly inhibit or kill algae in the water body; the other is to control the concentration of algae cells and their secretions in the effluent at water treatment plants to meet the standards for drinking water hygiene. The commonly used methods to address algal blooms include chemical, biological, and physical methods. Chemical methods primarily rely on chemical agents to harm algae [7], such as halogens, hydrogen peroxide, potassium permanganate, and ozone, which possess oxidizing properties and can effectively kill blooming algae [3], [39], [46]. Biological methods use higher plants and aquatic organisms to inhibit algae growth [47]. Physical methods mainly include filtration, flotation, and ultrasound techniques [48].

Ultrasonic technology, characterized as an advanced oxidation process, has found extensive reports in environmental treatment, and in the realm of algal bloom management, the instantaneous cavitation effect of ultrasound can generate powerful shear forces, effectively destroying algal cells and ensuring a clean and efficient removal process [17], [30], [33], [40]. This method is not only more environmentally friendly and efficient than traditional chemical and biological treatments but also addresses the issues of high costs and low efficiency associated with conventional physical methods. For example, Hao et al. utilized low-power high-frequency (1.7 MHz) ultrasound to remove the *Chlorophyll* in algae, leading to a significant reduction in total algal toxins in the water post-treatment [13]. Similarly, Fan et al. achieved a remarkable algal removal rate of 64.1 % under optimal conditions of 45 kHz ultrasonic frequency, 60 W input power, and a 48-second exposure time [9]. Xu et al. reported a substantial decrease in algal cell density by 33.49 % following low-frequency ultrasound irradiation at 29.4 kHz for 15 min, noting a more pronounced decline in the early stages of treatment when compared to the control group [41]. The mechanism of ultrasonic action on algal cells manifests through two primary pathways: cell membrane disruption and oxidative stress. Cavitation induced by ultrasonic waves creates microbubbles that undergo periodic changes and may even eventually explode, producing high temperatures and pressures capable of compromising the integrity of algal cell membranes, potentially resulting in cell death or damage. Additionally, these waves can generate reactive oxygen species (ROS), which induce oxidative stress and damage cellular components within the algal cells.

Despite these promising results, the majority of current research concentrates on the immediate or short-term effects of ultrasound on algae, with limited exploration into the long-term inhibitory impacts. Tang et al. discovered that treating *Arthrospira platensis* with ultrasound every 3 days for 4 min yielded an algal inhibition rate of 30.1 % [32]. Huang et al. furthered this research by conducting two ultrasound treatments at 20 kHz and 0.0025 W/mL, each lasting 1 min with a 36-hour interval, which resulted in an algal removal rate of 86.7 % with notably lower energy consumption [16].

Furthermore, due to the ubiquity of cyanobacteria worldwide, most research has focused on *Microcystis aeruginosa*. For example, Purcell et al. achieved removal rates of 16 ± 2 % and 20 ± 3 % for different algal cells at a frequency of 862 kHz, with ultrasound energy densities of 133 and 67 kWh/m^3^, respectively [27]. Shi et al. demonstrated that ultrasound can significantly remove *Microcystis aeruginosa*, but it is ineffective against *Aphanizomenon flosaquae* [31]. It is worth noting that different algae have varying degrees of resistance to ultrasound due to differences in their physiological structures. Duan et al. compared the effects of ultrasound on *Microcystis aeruginosa* and *Chlamydomonas*, finding that ultrasound destroyed the tightly bound single-electron acceptor in *Microcystis aeruginos*a and blocked the electron flow in the photosystem II, while *Chlamydomonas* showed stronger resistance to ultrasound [8]. The effects of ultrasound on different species of algal cells still need to be clarified.

Previous studies have shown that ultrasound can remove algae from water, inhibit their growth and photosynthesis, with most research focusing on the immediate or short-term effects of ultrasound on cells, but there is little research on the long-term inhibitory effects of ultrasound on algae. Therefore, it is necessary to further elucidate the potential mechanisms of ultrasound on algae, particularly at the level of related gene expression. After ultrasound treatment, if the culture conditions are appropriate, algal cells can recover their vitality and continue to divide and proliferate [27], so it is also important to study the physiology of algae within a few days after ultrasound treatment. Moreover, in naturally eutrophic waters with severe algal blooms, there are various types of algae from different phyla, each with different responses to ultrasound, making it necessary to study the responses of various algae in mixed algal communities.

**Scheme 1:**
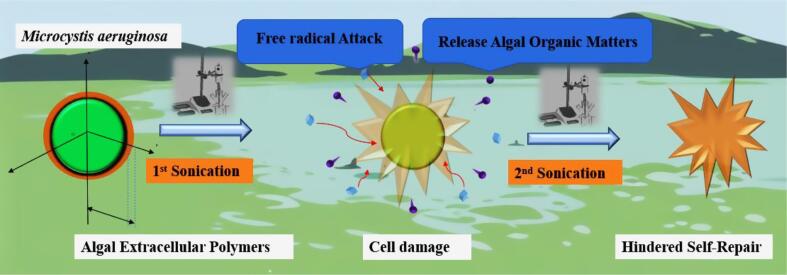
Illustration of *Microcystis aeruginosa* cells undergoing oxidative stress after two rounds of ultrasonic treatment.

Accordingly, this study investigates how different types of algae react to ultrasound when they are in a mix algal suspension containing *Microcystis aeruginosa*, *Lacustrine Oocystis, Chlorella vulgaris*, and *Navicular*. Since low frequencies often require less energy to achieve the same cavitation effects compared to higher frequencies, making them more sustainable for large-scale applications, a commercial 80 kHz bath was selected for the treatment. Aimed at the long-term inhibition of algae after ultrasound treatment, this research utilizes analytical methods to study the changes in the physiological characteristics of algal cells after ultrasound treatment in the fields of cytology, molecular biology, and genetics. This work further explores the mechanism of action of ultrasound treatment on *Microcystis aeruginosa* (Scheme 1), which has important theoretical significance and practical needs for the treatment of actual eutrophic waters. Our results showed that low-frequency ultrasound can Inhibit the growth of cyanobacteria beyond the treatment period, preventing rapid regrowth. Most field trials of ultrasound for controlling cyanobacterial blooms in expansive water bodies employ brief treatment durations. Detailed analysis of such short-term exposures on target blooms and co-existing beneficial algae could provide a reference for parameter optimization studies. Thus, by bridging the gap between short-term efficacy and long-term practicality, the study provides a foundation on ultrasonic algal bloom control.

## 2 Materials and methods

### 2.1 Microalgal species

*Microcystis aeruginosa* (strain FACHB-315) was obtained from the Chinese Academy of Sciences, while *Lacustrine Oocystis, Chlorella vulgaris, and Navicular* were acquired from a fisheries store on Taobao. These algae were cultured at a controlled temperature of 25 ± 0.5 °C under incandescent light at an intensity of 2000 lx, with a light–dark cycle of 12 h each (12 h light, 12 h dark) in BG11 and CSI media. All experimental procedures were conducted using cells in the stable growth phase, with algae concentrations reaching approximately 10^7^ cells/mL. To simulate the situation of cyanobacteria bloom, the experiments utilized a mixed algal sample consisting of cyanobacteria (dominantly Microcystis aeruginosa, 85 % relative abundance), *Oocystis lacustris*, *Chlorella vulgaris*, and *Navicula* species each constituted 5 % of the algal population.

### 2.2 Ultrasonic treatment

As 80 kHz cleaning equipment is widely available and commonly used in both industrial and research settings, it offers a practical and cost-effective solution for our study. In this work, the ultrasonic cleaner is featured with a rated frequency of 80 kHz, an output power of 240 W, and a capacity of 10.8 L. To conduct the experiments, the algae mixture (100 mL) is placed in a container, which is then positioned within the ultrasonic cleaner. The ultrasonic power density was determined through calorimeter measurement [19], [43], which quantifies the power dissipated into the liquid by the ultrasound. This method yielded an ultrasonic power density of 1.5 W/mL. The algal suspension underwent ultrasonic treatment for 20 min, followed by a 5-day storage period, after which samples were collected and analyzed. For selected trials, an additional 15-minute ultrasound exposure was administered to the algal suspension 2.5 days post-initial treatment. Solutions for algae counting and physicochemical analysis were extracted. Each experiment was replicated three times to ensure reliability and consistency of the results.

The generation of free radicals under ultrasonic irradiation was detected using the colorimetric reaction between KI and free radicals. In this method, free radicals react with iodide ions (I^-^) from KI, resulting in the formation of iodine (I_2_) [1]. The appearance of iodine can be visually detected using a spectrophotometer. 0.5 mL of the reaction solution was mixed with 0.2 mL of 0.1 M KI solution, followed by 30 s of ultrasonic treatment. The absorbance of the solution was then measured at a specific wavelength of 352 nm using a UV–Vis spectrophotometer.

### 2.3 Analysis methods

#### 2.3.1 Determination of growth inhibition rate

After ultrasound treatment, the supernatant was carefully collected using a pipette, and 0.1 mL of the sample was taken for microscopic examination and counting. Under the microscope, algae larger than 1.5 μm were identified and counted. The counting process repeated three times for each sample. The average value was recorded as the algal cell count for that sample. The growth inhibition rate ηI was calculated using Equation (1).

(1) $$\eta_{I}=\frac{\left(C_{c}-C_{t}\right)}{C_{0}}\times 100\%$$

wherein, ηI represents the growth inhibition rate, C_c_ denotes the control group, and C_t_ indicates the absorbance value of the experimental group at a specific time point.

#### 2.3.2 Determination of chlorophyll *a*, phycobiliprotein, UV254, and antioxidant enzyme activities

For chlorophyll measurement, the total volume of the sample (100 mL) was filtered using a glass fiber filter (GF/F). The filter cake and filter paper were collected and then placed in a test tube containing 10 mL of a 90 % acetone aqueous solution to extract the pigments. The test tube was kept in the dark at 4 °C for 24 h. Subsequently, the tube was centrifuged at 10,000 × g for 10 min to separate the paper and cellular residues. Three drops of 100 mN HCl were added to the extract, and the absorbance was measured at λ = 649 nm, λ = 665 nm, and λ = 750 nm. The spectrophotometer was zeroed at 750 nm to correct for turbidity and contaminating colored substances. [28]. The concentration of *chlorophyll* in each sample was calculated using Equation (2) [14].

(2) $$\mathrm{chla}=13.95{\mathrm{OD}}_{665}-6.88{\mathrm{OD}}_{649}$$

The total phycochrome protein (PB) is calculated as the sum of phycocyanin (PC), allophycocyanin (APC), and phycoerythrin (PE). Following this, the algal cell suspension is centrifuged at 4,500 rpm for 10 min, and the supernatant is then discarded. The algae cells are washed twice with phosphate-buffered saline (PBS). For the freeze–thaw process, the samples are frozen at −80 °C for 1 h and then thawed in the dark at room temperature. The supernatant is collected through centrifugation to measure its optical densities at 565 nm (OD_565_), 620 nm (OD_620_), and 650 nm (OD_650_). The concentration calculation formula for phycocyanin (PC), phycoerythrin (PE), and allophycocyanin (APC) is as follows [37], [24].

(3) $$PC\left(mg/L\right)=\frac{{\mathrm{OD}}_{620}-0.7\times {\mathrm{OD}}_{650}}{7.38}$$

(4) $$APC\left(mg/L\right)=\frac{{\mathrm{OD}}_{650}-0.19\times {\mathrm{OD}}_{620}}{5.65}$$

(5) $$PE\left(mg/L\right)=\frac{{\mathrm{OD}}_{565}-2.8\times PC-1.34\times APC}{12.7}$$

The UV absorbance at 254 nm (UV_254_) can represent the organic matter content in the solution. Therefore, after filtering the algal suspension with a 0.45 μm membrane, the changes in UV_254_ of the filtrate at specific time points were measured using a UV–Vis spectrophotometer (JINGHUA-752).

The Malondialdehyde (MDA), Catalase (CAT), and Superoxide Dismutase (SOD) concentrations were determined using the appropriate detection kits, which were purchased from the Jiancheng institute of biological engineering in Nanjing, China.

#### 2.3.3 Fluorescence excitation-emission matrix (EEM) spectra

The excitation-emission matrix (EEM) spectra of all samples were measured using a fluorescence spectrophotometer (F-4500, Hitachi, Japan). The EEM spectra were collected alongside corresponding scanning emission spectra, ranging from 250 nm to 550 nm with 10 nm increments, by adjusting the excitation wavelength from 20 nm to 500 nm in 10 nm intervals. Data analysis was performed using Origin Pro 2021, and the results were presented as contour lines.

#### 2.3.4 Scanning electron microscopy (SEM) and transmission electron microscopy (TEM) imaging

We used scanning electron microscopy (SEM) and transmission electron microscopy (TEM) to analyze the morphological transformations that the algal cells underwent after treatment. Samples were prepared by centrifuging at 10,000 rpm for 10 min to obtain cell pellets. These pellets were then fixed in a 2.5 % glutaraldehyde solution for 12 h at 4 °C to preserve their structural integrity. Following fixation, the samples were washed three times with phosphate-buffered saline (PBS) to remove any excess fixative. Subsequently, dehydration was achieved through a series of ethanol concentrations (10 %, 30 %, 50 %, 70 %, 80 %, and 90 %) to eliminate water content.

### 2.4 Transcriptome analysis

RNA sequencing was carried out on algal samples from both the control group and the ultrasound-treated group (exposed to ultrasound for 20 min). Total RNA was extracted, and its quantity was assessed using the Illumina HiSeq 2000 platform. RNA samples from both groups, with an OD_260_/OD_280_ ratio of ≥1.8, were sequenced on the Illumina platform, generating image files. These files were then converted into raw data (Raw Data) in FASTQ format using the platform's proprietary software. To ensure data quality, raw reads with adapters at the 3′ end, low-quality sequences, and those with more than 10 % unknown bases were removed, resulting in clean reads. These clean reads were subsequently meticulously mapped to the reference genome and gene sequences to calculate comparative ratios. The expression levels of individual genes were normalized, and the fragments per kilobase of transcript per million mapped reads (FPKM) were calculated for each segment. Generally, genes with an FPKM value greater than 1 were considered to be expressed. For differential gene expression analysis, the DESeq method was employed. Genes were identified as differentially expressed if they met the criteria of an expression fold change of |log2FoldChange| > 1 and a significance level of P < 0.05.

## 3 Results and discussion

### 3.1 Inhibition experiment of algae mixture

Researchers have studied the effects of single ultrasonic treatments on the concentration of algal cells. However, research into the following changes and persistent effects on algal cell populations post-treatment is comparatively limited. We assume that a short period of ultrasound can cause some kind of stress to algal cells. Then, if the ultrasonic treatment is carried out again, can the concentration of algal cells be further controlled? This study has reference significance for the ultrasonic control of cyanobacteria blooms.

**Fig. 1:**
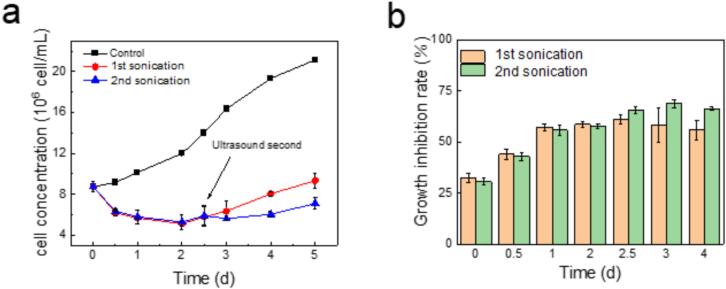
Inhibition experiment of algae supernatant (80 kHz, 0.15 W/mL). (a) Cell concentration; (b) Growth inhibition rate. The initial treatment consisted of a single 20-minute session on the first day. A second 20-minute session was conducted on the 2.5th day for the second ultrasound group.

Fig. 1 shows that ultrasonic treatment has a significant impact on the growth of mixed algal cells (Fig. 1a), as reflected by the observed inhibition rates (Fig. 1b). After a 20-minute ultrasonic treatment, the growth inhibition rate reaches 57.8 % on the first day and begins to recover gradually from the 2.5th day. By the 5th day, the cell concentration remains lower than that of the control group, and the growth inhibition rate remains around 50 %, indicating that a single ultrasonic treatment has already had an inhibitory effect on the mixed algal population.

The second ultrasonic treatment is administered at the critical recovery time point, which is the 2.5th day. After the treatment, the cell density is not only lower than that of the control group but also lower than that of the group that received only one ultrasonic treatment. By the 3rd day, the inhibition rate has further increased to 68.7 %. The continuous decrease in cell concentration over time highlights the long-lasting inhibitory effect of ultrasonic treatment on algal growth. This observation validates the effectiveness and durability of using two 20-minute ultrasonic treatments to suppress algal proliferation. For the mixed algal suspension, the cell counting data indicate that despite undergoing two rounds of ultrasonic treatment, the algal cell count continues to exhibit a gradual recovery trend. Compared with the experimental results of a single ultrasound treatment, the trend of algal cell regrowth has been further slowed down.

### 3.2 Chlorophyll-a and phycobiliproteins change of algae mixture

**Fig. 2:**
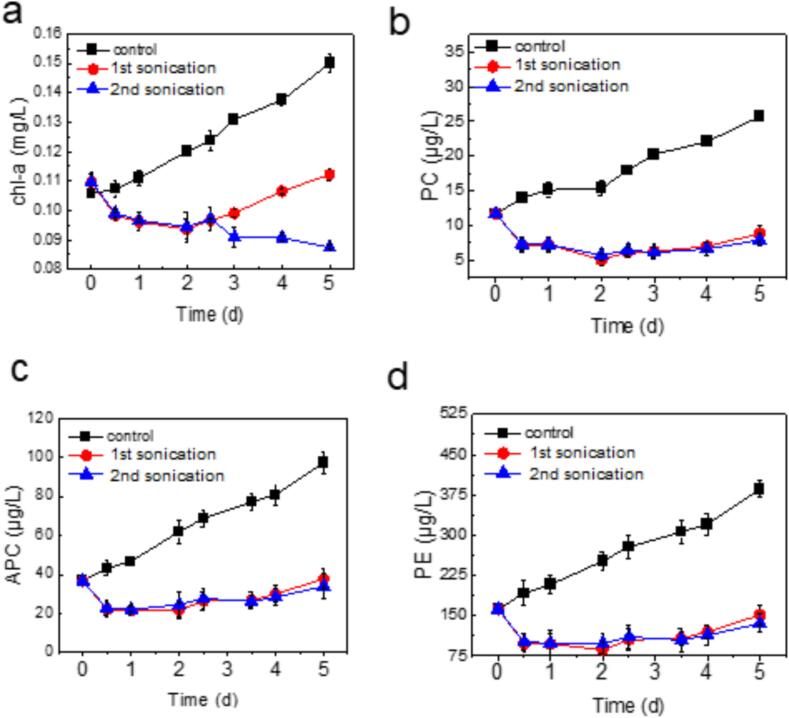
Concentration of photosynthetic pigments in the algae supernatant. (a) Chl-a (Chlorophyll *a*); (b) PC (Phycocyanin); (c) APC (Allophycocyanin); (d) PE (Pheophytin). The mixed algal suspension was cultivated for a period of 5 days. The treatment was conducted at a frequency of 80 kHz and an intensity of 0.15 W/mL. The first ultrasound treatment consisted of a single 20-minute session on the first day, and the second treatment involved a subsequent 20-minute session on the 2.5th day, following the initial treatment.

Photopigments are crucial for the absorption and utilization of light, profoundly influencing the primary photosynthetic reactions within algal cells. To understand how different photopigments respond to ultrasonic treatment, we continuously monitored pigment changes following both single and double treatments. Fig. 2(a) reveals that a single ultrasonic treatment led to a substantial decrease in chlorophyll *a* concentration, from 0.16 mg/L to 0.09 mg/L. After the cells underwent self-recovery and reproduction, the chlorophyll *a* content in the single treatment group gradually rose, while it continued to fall in the double treatment group. This difference might be due to the disruption of the photosynthetic system and the fact that some cells, although in a decline phase, had not yet completely died [10]. Moreover, reactive oxygen species (ROS) produced by ultrasonic waves could attack chlorophyll, converting some chl-b into chl-a, thus increasing the relative chlorophyll *a* content in the cells.

It should be noted that in the mixed algal solution of this experiment, phycocyanin was primarily from *Microcystis aeruginosa*, since phycocyanin is a distinctive blue pigment-protein complex found in cyanobacteria and certain red algae. Fig. 2(b) shows that phycocyanin was highly sensitive to a single ultrasonic treatment, but the response to the second treatment session was less pronounced. Fig. 2(c) depicts the changes in allophycocyanin levels. Allophycocyanin are compounds derived from the degradation of chlorophylls. Both experimental groups exhibited consistently higher allophycocyanin levels than the control group, with the double treatment group reaching a peak of 0.37 mg/L on the 5th day. This indicates that under ultrasonic stimulation, chlorophyll may undergo various oxidation reactions, potentially converting into allophycocyanin and accumulating significantly, suggesting that the algae are in a declining phase. Fig. 2(d) illustrates the changes in phycoerythrin levels. Pheophytin are important high-value substances in microalgae, playing a key role in reducing lipid peroxidation and maintaining cell stability. Following ultrasonic treatment, both groups showed a decrease in phycoerythrin content. The single treatment group experienced a slightly increase in phycoerythrin content before the 2.5th day, indicating an enhanced ability to withstand external stress and a subsequent increase in algal cell numbers (as shown in Fig. 1). This study's systematic analysis of pigment content within the photosynthetic system demonstrates that ultrasonic treatment has a significant inhibitory effect on algae, particularly by directly regulating the photosynthetic process, effectively suppressing the growth of algal cell numbers.

It is known that when ultrasonic waves propagate through a liquid, they generate microbubbles that rapidly expand and collapse under the influence of the sound waves, creating localized high-temperature and high-pressure environments. These extreme conditions can trigger a series of chemical reactions, including the generation of free radicals. In sonochemistry, the production of free radicals primarily occurs during the collapse of cavitation bubbles. During bubble collapse, localized temperatures can reach several thousand degrees Celsius, and pressures can rise to hundreds of atmospheres. Under such extreme conditions, water molecules (H_2_O) undergo thermal decomposition, yielding hydroxyl radicals (·OH) and hydrogen radicals (·H). The chemical reaction is represented as follows:

(6) H_2_O → H· + OH·

(7) OH· + OH·→ H_2_O_2_

The experiment proves that the 80 kHz ultrasonic radiation can generate active radicals within 30 s (Fig. S1). These free radicals exhibit high reactivity and can readily engage in reactions with other molecules. For instance, hydroxyl radicals (·OH) can undergo addition reactions with double bonds or aromatic rings within chlorophyll molecules, leading to structural modifications that subsequently alter their light absorption properties (as depicted in Reaction (8). Furthermore, hydroxyl radicals can degrade chlorophyll molecules through oxidative processes, resulting in the breakdown of their molecular structure. This process may involve multiple steps, ultimately leading to the formation of small molecular compounds (as shown in Reaction (9). Consequently, hydroxyl radicals can disrupt the porphyrin ring structure of chlorophyll through either addition reactions or oxidation, thereby affecting its light absorption capacity and stability.

(8) Chlorophyll+·OH→Chlorophyll

(9) Chlorophyll + ∙OH → Oxidation products + Other by-products

### 3.3 Cell integrity of algae supernatant

**Fig. 3:**
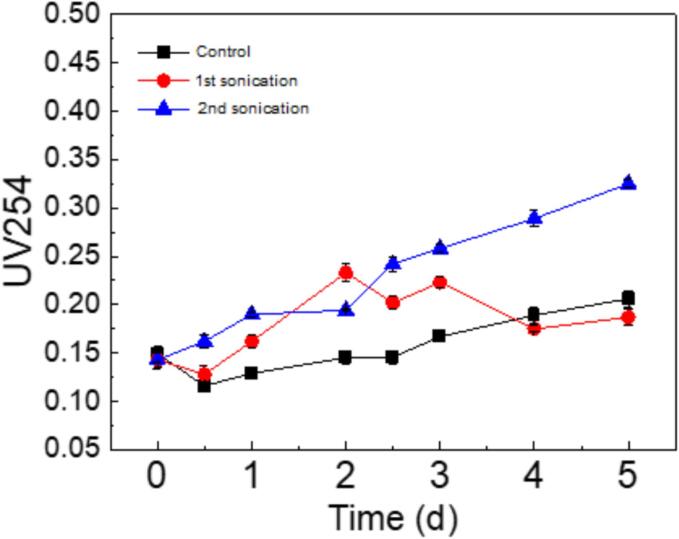
UV_254_ variation of algae supernatant during 5 days. The treatment was conducted at a frequency of 80 kHz and an intensity of 0.15 W/mL. The single ultrasound treatment consisted of a 20-minute session on the first day. The second ultrasound treatment involved a subsequent 20-minute session on the 2.5th day, following the initial treatment.

Fig. 3 shows that after a single ultrasonic treatment, the UV_254_ absorbance of the mixed algal solution initially increased and then decreased. Given the rise in UV_254_ and the relatively stable cell concentration from day 1 to 3, the initial increase in UV_254_ is attributed not only to the rapid release of intracellular organic matter (IOM) during cell lysis but also to the gradual secretion of extracellular organic matter (EOM) under oxidative stress stimulation [34]. This observation is further supported by the analysis of fluorescently labeled extracellular products using excited emission microscopy (EEM). The stability of UV_254_ from the 3rd day onwards, which coincides with cell recovery and rapid proliferation, suggests a balance between the release and reuse of organic matter during the recovery process [35], [49]. When a second ultrasonic treatment was applied on the 2.5th day, UV_254_ concentrations rose rapidly. This rapid increase further confirms the leakage of IOM due to the disruption of algal cells and the additional production of EOM stimulated by the ultrasonic treatment. The measurement of UV_254_ in the supernatant indicates that the ultrasonic treatment damaged the integrity of the algal cell walls and membranes, thereby compromising the structural integrity of the cells.

**Fig. 4:**
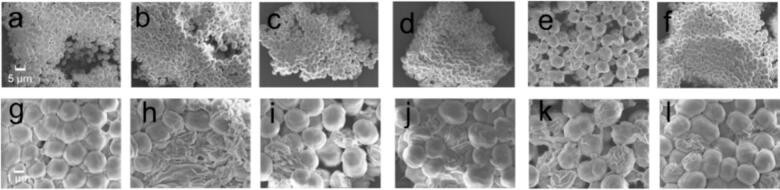
SEM images of algae before and after sonication at two different magnifications: 10 k (subfigures a to f) and 30 k (subfigures g to l). These images depict the algae at various stages: (a, g) prior to treatment, (b, h) immediately following a 20-minute ultrasound session, (c, i) at 2.5 days post-treatment, (d, j) after the second ultrasound application, (e, k) at 3 days, and (f, l) at 5 days.

Fig. 4 presents a comparative SEM image analysis of the mixed algae before and after ultrasonic treatment. Pre-treatment images show algae cells that are tightly packed, with well-defined contours, maintaining a regular spherical shape and a smooth surface, and showing minimal signs of cellular damage. After undergoing a 20-minute ultrasonic treatment, the cells exhibited extensive surface wrinkling and were covered with a substantial amount of cellular debris and secretions. By the 5th day, the algal cells’ physiological vitality showed a resurgence, with an increase in cell count as their membrane integrity was gradually restored, and their surfaces returned to a smooth, undamaged state. These SEM images emphasize the effect of ultrasonic treatment on the structural integrity of algal cells. They also illustrate the algae's resilience, demonstrating that under favorable culture conditions, the cells can recover their metabolic activity after being stressed by ultrasonic waves.

### 3.4 EEM of IOM & EOM of algae mixture

Monitoring extracellular organic matter (EOM) is essential in both algal ecological research and the assessment of aquatic environmental quality. This study employed Excitation-Emission Matrix (EEM) technology to thoroughly analyze the characteristics of EOM in the non-ultrasonic control group, the single ultrasonic treatment group (20 min), and the second ultrasonic treatment group (two 20-minute treatments). EOM is not only a direct byproduct of algal metabolic processes but also serves as an indicator of algal growth status, metabolic activity, and the ability to adapt and respond to environmental stress. By carefully analyzing EOM, we can gain valuable insights into the physiological and ecological properties of algae, thereby improving our understanding of their sensitivity to ultrasonic radiation.

**Fig. 5:**
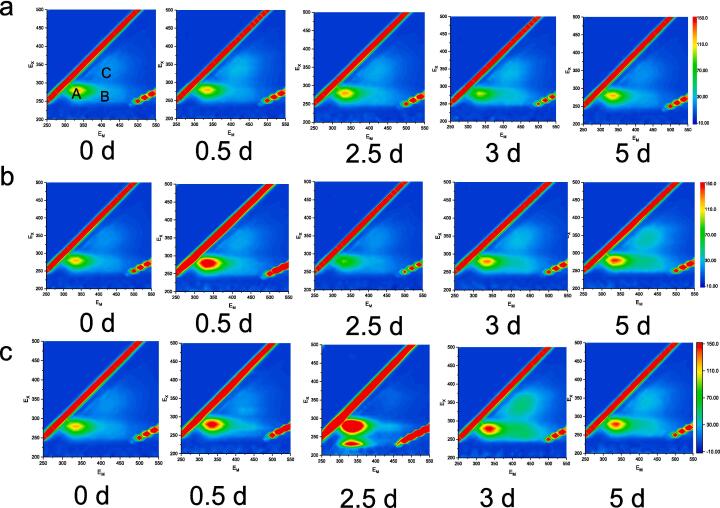
Excitation-Emission Matrix (EEM) of Extracellular Organic Matter (EOM) Changes. (a) Control; (b) Single ultrasound treatment; (c) Second ultrasound treatment. The single ultrasound treatment consisted of a 20-minute session on the first day. The second ultrasound treatment involved a subsequent 20-minute session on the 2.5th day, following the initial treatment.

Fig. 5(a) shows that the EOM in the control group is primarily composed of soluble microbial products and humic acid components. The control group's EOM exhibits three distinct characteristic peaks at excitation/emission wavelengths of 280/344 nm (Peak A, soluble microbial products), 333/415 nm (Peak B, humic acids), and 327/410 nm (Peak C, humic acids) [2], [15], [23], [25]. Notably, the humic acid components have higher peak intensities, and their content remains relatively stable throughout the cultivation period. Fig. 5(b) reveals that the EOM in the algae solution after 20 min of ultrasonic treatment is predominantly made up of soluble microbial products and humic acids. The fluorescence intensity of soluble microbial products initially decreases and then increases, while the peak intensity of humic acids continuously increases during cultivation. This increase in fluorescence intensity is attributed to both the increased secretion of EOM due to the rising cell count and the algae's response to external stress [23]. Fig. 5(c) illustrates that the EOM after the second ultrasonic treatment is mainly composed of aromatic proteins, soluble microbial products, and humic acids. The peak intensity of humic acids initially increases and then decreases. On the 0.5th day post-ultrasonic treatment, Peaks A and B significantly intensify, indicating a substantial release of aromatic proteins from damaged cells. The subsequent disappearance of these peaks suggests extensive cell death.

In the control group, which did not receive ultrasonic treatment, the intensity of Peak A remains consistent. However, following a single ultrasonic treatment, the intensity of Peak A experiences a two-phase change, initially decreasing and then increasing over the course of the ongoing cultivation period. After the second ultrasonic treatment, the intensity of Peak A (soluble microbial products) is notably reduced during the latter stages of cultivation. These findings suggest that the intensity of Peak A in the EEM can serve as a marker for microalgal activity. Conversely, a substantial decrease in the fluorescence intensity of Peak A is observed when the algal cells lose their viability. Moreover, in the control group, the fluorescence intensity of humic acids gradually increases. In contrast, after ultrasonic treatment, the fluorescence intensity of Peak B shows significant fluctuations, indicating that humic acids derived from algae are sensitive to ultrasonic radiation. The changes in these fluorescent substances and their correlation with the integrity and physiological activity of algal cells are worthy of further investigation.

**Fig. 6:**
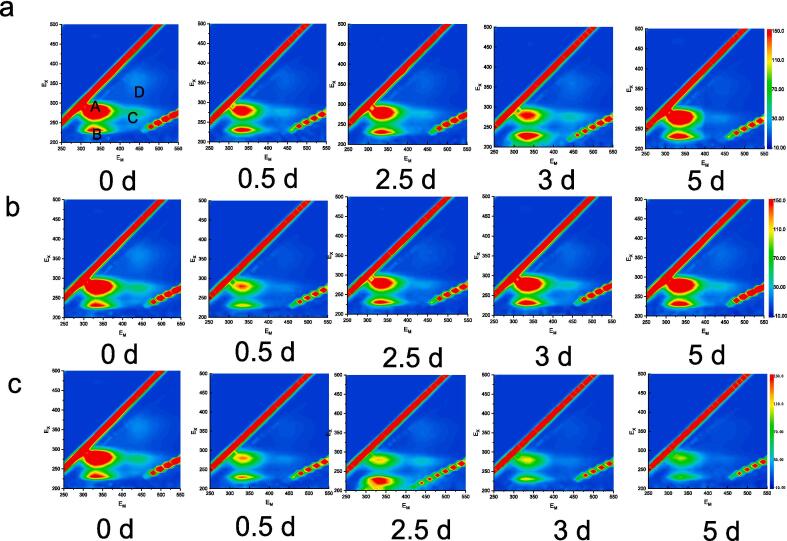
Excitation-Emission Matrix (EEM) of Intracellular Organic Matter (IOM) Changes. (a) Control; (b) Single ultrasound treatment; (c) Second ultrasound treatment. The single ultrasound treatment consisted of a 20-minute session on the first day. The second ultrasound treatment involved a subsequent 20-minute session on the 2.5th day, following the initial treatment.

Intracellular Organic Matter (IOM), the organic material found within algal cells, serves as a direct indicator of the cells' physiological state and overall health. Analyzing IOM allows for an assessment of the vitality, metabolic activity, and adaptability of algal cells to external environmental conditions. Fig. 6(a) illustrates that the IOM in the internal control group, which was not subjected to ultrasonic treatment, is predominantly composed of aromatic proteins, humic acids, and soluble microbial components. This group's IOM features four distinct characteristic peaks at excitation/emission wavelengths of 281/344 nm (Peak A, resembling soluble microbial products), 228/334 nm (Peak B, aromatic proteins), 272/445 nm (Peak C, resembling humic acids), and 360/445 nm (Peak D, resembling humic acids) [4], [5], [26], [42]. Among these, the humic acid-like substances within the cells have relatively lower peak intensities, while the content of soluble microbial substances, which includes extracellular polymers such as polysaccharides, proteins, nucleic acids, and lipids found predominantly near the algal cell membranes, is relatively high. Over time, there is no significant change in the content of these regions. For the single ultrasonic treatment group (20 min), Fig. 6(b) reveals that the IOM in this group is distributed mainly in regions I, II, IV, and V, consisting of aromatic proteins, humic acids, and soluble microbial components. Following a single ultrasonic treatment, the fluorescence intensity of IOM significantly decreases, indicating that IOM may have been released into the solution due to damage to the cell outer membranes, thereby increasing the external Extracellular Organic Matter (EOM). On the 0.5th day, the continuous decrease in the intensity of Peak A suggests that the metabolic processes of the cells may also have been affected. Aromatic proteins, as intracellular bioactive substances, indicate cell death when their levels decrease. As cultivation continues, the IOM content gradually increases during the cell growth. Fig. 6(c) depicts the IOM after double ultrasonic treatment, which is primarily composed of aromatic proteins, humic acid-like substances, and soluble microbial components. Compared to the control group without ultrasonic treatment, the fluorescence intensity of these components is weaker. During continuous cultivation, the fluorescence intensity of Peak A transitions from weak to strong, while Peaks B and C gradually increase. This change suggests that during cell apoptosis, the metabolic process may involve the conversion of soluble substances to humic-like substances. In recent years, humic substances in natural waters have been found to participate in important geochemical processes such as photocatalysis and iron cycling. Therefore, the impact of humic substances released from algae by ultrasonic treatment on the environment is a topic that warrants further investigation.

This study elucidates the profound physiological and metabolic responses of algal cells to ultrasound stress through a detailed analysis of changes in extracellular organic matter (EOM) and intracellular organic matter (IOM) fluorescence. The subsequent increase in EOM fluorescence intensity after ultrasound treatment signifies the release of cellular contents, presumably due to membrane damage, which includes the discharge of soluble microbial products and humic acids, both of which serve as indices of cellular stress and injury. The modifications in the IOM fluorescence patterns, particularly the biphasic changes in Peak A intensity and the fluctuations in humic acid-like substances, underscore the dynamic and complex nature of intracellular responses to ultrasound exposure. These changes imply disruptions within the cells, a heightened secretion of stress-related substances, and the potential for cell death. Our research underscores the role of ultrasound as a potent stressor for mixed algal cells, affecting their growth, structural integrity, photosynthetic capacity, and biochemical composition. Insight into these stress mechanisms is critical for evaluating the potential applications and environmental consequences of ultrasound in algal management and water treatment processes. This study introduces an innovative dimension to EEM analysis by concentrating on the detailed changes in EOM and IOM fluorescence to assess the cellular responses to ultrasound stress. It emphasizes the necessity for investigating the long-term effects and adaptive strategies of algal cells in the face of ultrasound exposure. Notably, the study's methodology, which includes a 5-day cultivation period following ultrasound treatment and a subsequent second ultrasound treatment at the 2.5-day mark a methodological approach that has not been previously reported, which enables a comprehensive understanding of the algal response to ultrasound stress.

### 3.5 Antioxidant system of algae after ultrasonic treatment

**Fig. 7:**
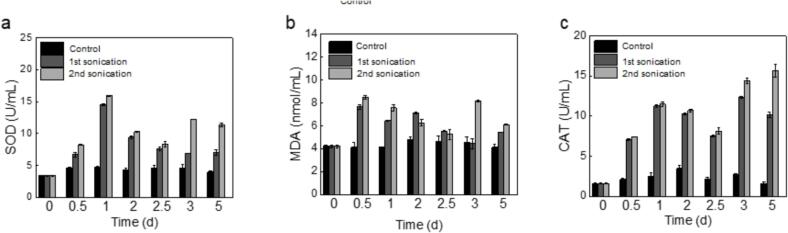
The changes in Superoxide Dismutase (SOD), Malondialdehyde (MDA), and Catalase (CAT) levels. The cultivation period for the mixed algal suspension was 5 days. The treatment was conducted at a frequency of 80 kHz and an intensity of 0.15 W/mL. The single ultrasound treatment consisted of a 20-minute session on the first day. The second ultrasound treatment involved a subsequent 20-minute session on the 2.5th day, following the initial treatment.

Superoxide dismutase (SOD) and catalase (CAT) are crucial antioxidant enzymes that are in charge of managing oxidative stress within algal cells. SOD's primary function is to counteract the toxicity of free radicals, while CAT is essential for the elimination of hydrogen peroxide, a harmful byproduct of cellular metabolism [20], [44]. Malondialdehyde (MDA), a product of lipid oxidation in cell membranes, is a toxic substance that can disrupt membrane function [11]. In Fig. 7, initial exposure of algal cells to ultrasound leads to a sharp increase in oxidative stress, which peaks on the first day and then starts to fall back on the second day. After a single ultrasound treatment, the level of SOD remained higher than that of the untreated control group. For the experimental samples that received a second ultrasound treatment on the 2.5th day, their SOD levels increased by the 3rd day, compared to both the control group and the group that received only one ultrasound treatment. This suggests that the algal cells sustain additional damage from the secondary ultrasound exposure, resulting in a higher oxidative stress level. It should be noted that following the second ultrasound treatment, MDA levels continue to rise before decreasing on the 5th day. The excessive accumulation of antioxidants in the cells can lead to changes in membrane permeability, initiate oxidative damage to biomolecules, and produce lipid peroxidation-related signaling molecules, potentially leading to cell dysfunction and death. The data indicate that CAT exhibits greater resistance to ultrasound compared to SOD and MDA. This is consistent with the observed changes in cell integrity depicted in Fig. 4. The overall dynamic responses of SOD, CAT, and MDA in algal cells under ultrasonic stress provide a comprehensive understanding of the cellular antioxidant defense mechanisms and the extent of oxidative damage. These findings highlight the intricate relationship between oxidative stress, antioxidant enzyme activity, and cell membrane integrity in the context of ultrasonic exposure, offering valuable insights for assessing the impact of ultrasound on algal cell health and viability.

### 3.6 Ultrasonic treatment response in Microcystis aeruginosa: Differential gene expression analysis

Considering the pronounced sensitivity of *Microcystis aeruginosa* to ultrasonic treatment, we undertook a comprehensive differential gene expression analysis. It is performed to identify the genes that are upregulated or downregulated in response to the treatment. This helps to explain the molecular changes that occur within the algae and provides insights into the physiological and biochemical processes that are affected by the ultrasound. Utilizing the Illumina HiSeq 2000 platform, we analyzed gene expression levels for each sample, yielding raw data that included sample names, Q30 metrics, and percentages of ambiguous and accurately sequenced bases (Q20 at 99 % accuracy, and Q30 at 99.9 % accuracy). These data are summarized in Table 1. The analysis yielded over 15 million reads for subsequent assembly and analysis. These reads were then assembled and analyzed to identify differentially expressed genes (DEGs), which are genes whose expression levels change significantly in response to the treatment. Moreover, the high number of reads generated by the Illumina HiSeq 2000 platform allows for a robust analysis of gene expression. The reads are assembled into contigs, which are sequences that represent the genome or a part of it. This assembly process is essential for identifying DEGs, as it allows for the comparison of gene expression levels across different samples.

**Table 1:** Raw data statistics.

Sample	Reads No.	Base(bp)	Q30(bp)	N(%)	Q20(%)	Q30(%)
M1(Control)	15,632,502	2,360,507,802	2,247,686,070	0.00	97.54	95.22
M2(Sonication)	15,566,100	2,350,481,100	2,239,055,018	0.00	97.34	95.26

The Q20 and Q30 metrics are key indicators of the quality and reliability of sequencing data, reflecting the percentage of bases sequenced with accuracies above 99 % and 99.9 %, respectively. In our study, both the control and ultrasonically treated samples demonstrated Q20 and Q30 percentages that exceeded 95 %, which is a strong signal that the sequencing data were of high quality and suitable for further analysis.

**Fig. 8:**
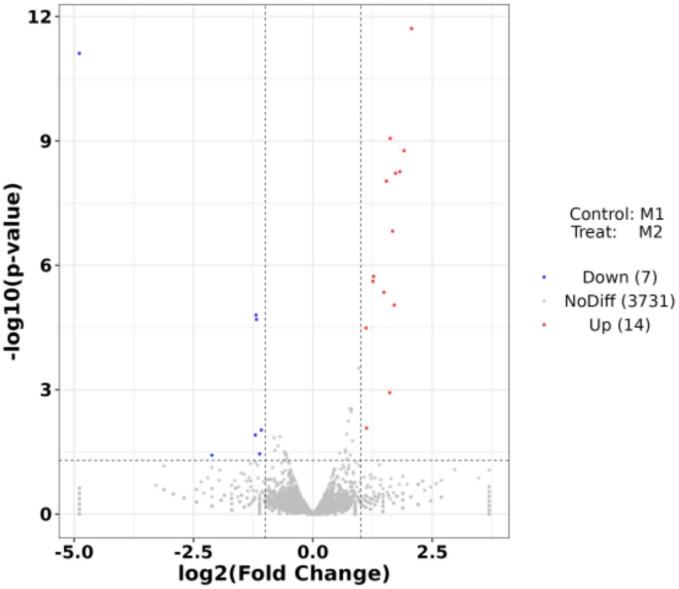
Volcano plot of differentially expressed genes (DEGs) in response to algal gene expression after 20 min of ultrasound treatment.

To visualize the genes that were differentially expressed (DEGs) following the ultrasonic treatment, we utilized a volcano plot, a powerful tool for data visualization (Fig. 8). The results from the 20-minute ultrasonic treatment revealed a total of 21 DEGs, with 14 genes showing increased expression (upregulated) and 7 genes showing decreased expression (downregulated). This detailed view of the gene expression changes provides a window into the molecular responses of *Microcystis aeruginosa* to ultrasonic stress. The volcano plot not only helps to identify DEGs but also allows us to assess the statistical significance of their changes. Genes that are downregulated in the “Teat” group but not significantly different from the control are located below the horizontal axis, while those upregulated in the Case group and significantly different from the control are above the axis. The distance from the vertical axis represents the fold change, with more distant points indicating greater changes in expression levels. To further analysis of DEGs following ultrasonic treatment and provide aids in understanding the molecular basis of the algal response, the enrichment analysis of differentially expressed genes (DEGs) using Gene Ontology (GO) terms is conducted as follows.

**Fig. 9:**
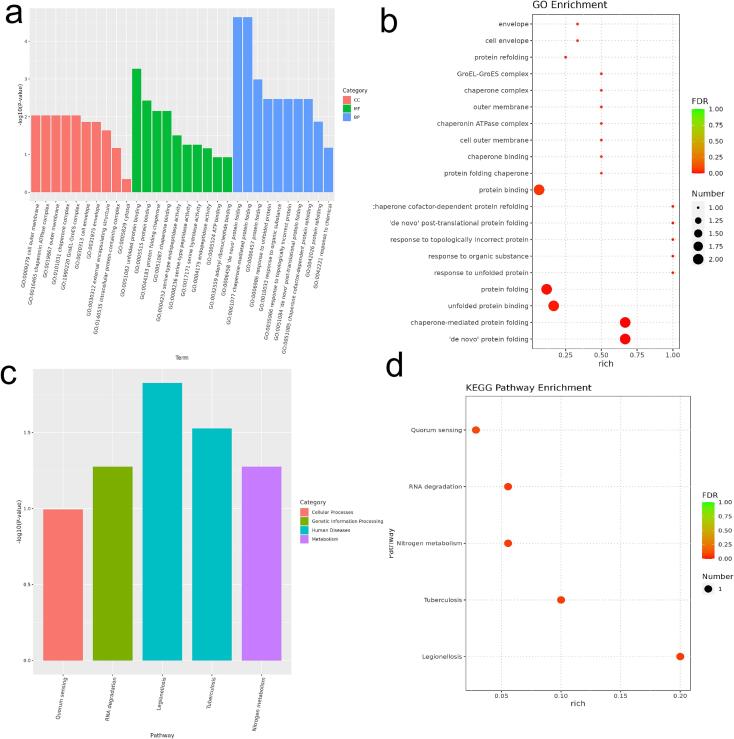
Response of *Microcystis aeruginosa* functional enrichment after 20-min ultrasound (a) Gene Ontology (GO) terms enrichment of DEGs showing the biological process, cellular component, and molecular function in ultrasound treatment; (b) GO enrichment analyzes bubble charts; (c) Kyoto Encyclopedia of Genes and Genomes (KEGG) pathways; (d): Kegg analyzes bubble charts.

By means of Gene Ontology (GO) analysis, we utilized the topGO enrichment analysis tool to annotate differentially expressed genes, calculating the number of genes associated with each GO term. We then employed the hypergeometric distribution method to calculate p-values (with a threshold for significant enrichment set at p-value < 0.05), thereby identifying GO terms that are significantly enriched compared to the whole genome background. These terms reveal the primary biological functions of the differentially expressed genes. Based molecular function (MF), biological process (BP), and cellular component (CC), we selected the top 10 GO terms with the lowest p-values or highest enrichment levels for presentation, as shown in Fig. 9 (a). Additionally, we selected the top 20 GO terms with the lowest False Discovery Rate (FDR) for presentation, as shown in Fig. 9 (b). The analysis results indicate that following ultrasound treatment, GO analysis revealed that in terms of cellular components (CC), the most significant genetic changes were observed in the extracellular membrane, unfolded protein binding, chaperone protein ATPase complex, outer membrane, and chaperone complex, all of which showed upregulated genes. These findings suggest that ultrasound not only causes mechanical damage to the algae but also triggers active repair processes, providing a possibility for the algae's reproduction and iteration. In terms of molecular function (MF), the changes in the heat shock protein GroEL-GroES were pronounced, and in terms of biological process (BP), the changes in protein folding complex and chaperone-mediated protein folding were most prominent, all showing upregulated genes. This indicates that ultrasound affects the physiological processes of *Microcystis aeruginosa* by influencing the protein folding process, thereby regulating the synthesis of related enzymes and proteins.

Based on the KEGG (Kyoto Encyclopedia of Genes and Genomes) pathway enrichment analysis of differentially expressed genes, we selected the top three pathways with the lowest p-values and highest enrichment levels for presentation, as depicted in Fig. 9(c). Additionally, we also presented the three pathways with the lowest False Discovery Rate (FDR) values, as shown in Fig. 9(d). The KEGG enrichment analysis revealed that upregulated genes are primarily involved in RNA degradation, while downregulated genes are associated with quorum sensing (QS) and nitrogen metabolism [36]. The proper processing, quality control, and turnover of cellular RNA molecules are crucial for various aspects of genetic information expression, and the ultrasound treatment promotes these processes, indicating that algae tried to replicate and express genetic information, as well as regulate cell growth and apoptosis in response to the external stress caused by ultrasound. The suppression of quorum sensing and nitrogen metabolism by ultrasound treatment suggests a significant alteration in the metabolic processes of algae under ultrasound treatment. Quorum sensing, a key factor in determining microbial interactions, enables microbial populations to receive signaling molecules, triggering a cascade of reactions and gene expression. When quorum sensing is inhibited, it affects the nutritional uptake of microorganisms in natural systems, subsequently impacting material and energy metabolism, thereby weakening the structure of microbial communities [18], [36], [45].

In summary, the KEGG pathway enrichment analysis has uncovered the multifaceted impact of ultrasound treatment on algal gene expression, including the enhancement of RNA degradation, the inhibition of quorum sensing, and the regulation of nitrogen metabolism. These changes suggest that algae tried to rapidly adapt to environmental changes by promoting RNA degradation after sonication, while simultaneously inhibiting quorum sensing to reduce algal aggregation and slowing down nitrogen metabolism to affect algal growth rate. These mechanisms, working together, may mitigate the formation of harmful algal blooms.

**Fig. 10:**
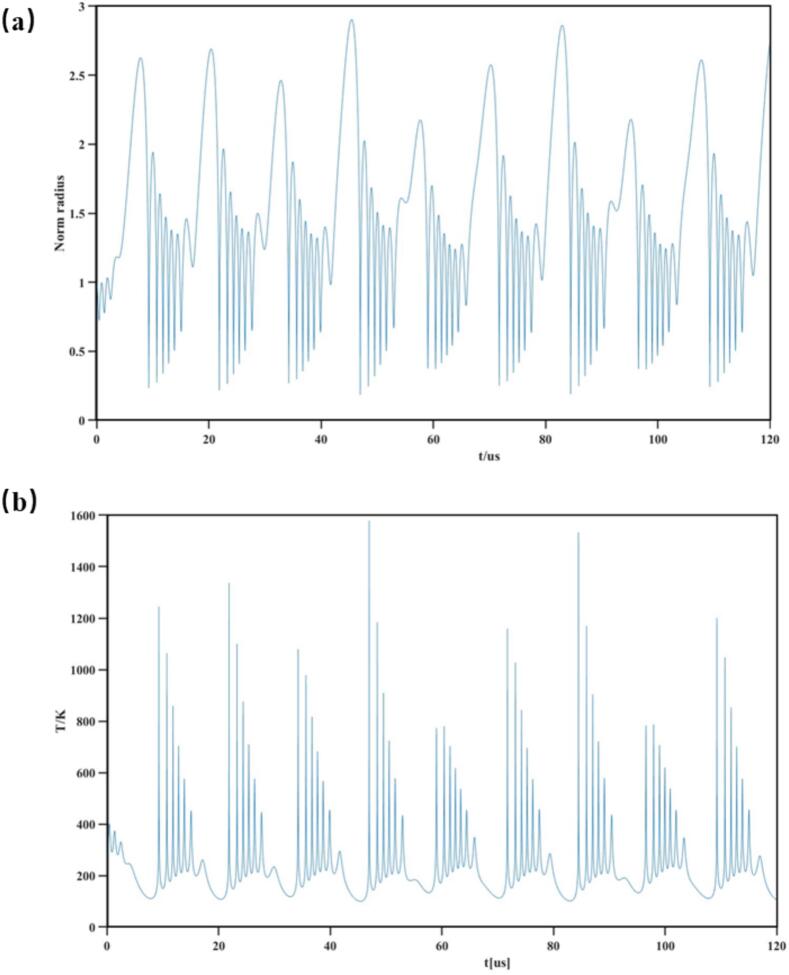
(a) Radius–time profile for a single bubble exposed to 80 kHz ultrasound at 1.5 W/mL. (b) The fitting of the temperature change of the cavitation bubble. [R0 = 5 μm].

This study demonstrates the application of ultrasonic treatment in algae control, with its mechanism primarily manifested through cavitation effects. Ultrasonic waves, characterized by their high-frequency acoustic properties, generate mechanical, thermal, and cavitation effects in the liquid medium, which work in synergy to cause damage to algae cells. We consider an ideal case of a single bubble at 80 kHz and 1.5 W/mL. The typical radius-time profile and numerically obtained temperature change is presented in the Fig. 10. The R/R_0_ is indicative of change in the volume of the bubble upon collapse. In the 80 kHz ultrasound field, compared with high-frequency ultrasound, cavitation bubbles have more time to grow. The process of cavitation bubble growth and collapse can be regarded as the movement process of the cavitation bubble wall. Our fitting shows that the volume of the cavitation bubble can cycle between 1 and 3 times its original volume, indicating that the mechanical force and mass transfer generated by the volume change of the cavitation bubble are objective. In addition, the fitting result of the temperature change of the cavitation bubble also shows that the maximum temperature can reach 1600 K. This is consistent with the test of the experimental group showing that the algal cells are subject to heat stress.

The mechanical effects of ultrasound can disrupt the cell walls and cell membranes of algae, leading to the leakage of cellular contents. When the acoustic intensity reaches a critical threshold, cavitation occurs, a unique phenomenon in the liquid medium. In an ideal liquid, molecules are strongly bonded with each other and exhibit high tensile strength. However, in actual liquids, the presence of microbubbles (cavitation bubbles) weakens this strength. As the alternating sound pressure produces negative and positive phases, the cavitation bubbles expand and subsequently collapse, leading to their rupture [6], [22], [38]. However, the research on how cavitation in the mixed algal suspension affects these algal cells is yet to be undertaken.

Research by Huang et al. [17], Rumyantsev et al. [30], Tao et al. [33], and Wu et al. [40] indicates that ultrasonic treatment effectively destroys algal cells through the instantaneous cavitation effect, ensuring a clean and efficient removal process. Currently, the algae removal rate of a single algae removal treatment fluctuates between 30 % and 90 %. It is very common for the algae removal efficiency of low-frequency ultrasound to be around 50 %. In this study, after 20 min of ultrasonic treatment, the cell concentration of mixed algae decreased by 57.8 %, and after the second treatment, this value further decreased to 68.7 %, indicating the cumulative effect of ultrasonic treatment, which can continuously inhibit algal growth. To gain a deeper understanding of the effects of ultrasonic treatment on algae cells, we conducted cell integrity analysis and EEM analysis. SEM images showed that after ultrasonic treatment, algae cells exhibited noticeable wrinkles, accompanied by the release of a substantial amount of cellular debris and secretions, indicating that the ultrasonic treatment had disrupted the cell walls and cell membranes of algae. EEM analysis further confirmed this, showing that the fluorescence intensity of soluble microbial products significantly decreased 0.5 days after treatment and then gradually recovered, suggesting that the ultrasonic treatment caused the leakage of cellular contents, followed by the beginning of the repair process of the cells. EEM analysis revealed that after ultrasonic treatment, there were changes in the fluorescence intensity of extracellular organic matter (EOM) and intracellular organic matter (IOM), which may reflect the metabolic and physiological responses of algae to ultrasonic stress. For example, an increase in the intensity of certain fluorescence peaks may indicate membrane damage and the release of cellular contents. Ultrasonic treatment can induce oxidative stress, leading to changes in the activity of antioxidant enzymes such as superoxide dismutase (SOD) and catalase (CAT). The increase in these antioxidant proteins may be an adaptive response of algae to oxidative stress, but excessive ultrasonic treatment may overload the antioxidant system and cause cell damage. The analysis of gene expression in *Microcystis aeruginosa* revealed that ultrasonic treatment led to an upregulation of genes related to RNA degradation and a downregulation of genes associated with quorum sensing and nitrogen metabolism. This suggests that ultrasonic treatment may affect the growth and metabolism of algae by regulating their RNA metabolism and nitrogen metabolism, providing an important molecular basis for the development of ultrasonic-based algae control strategies.

Compared to traditional chemical and biological treatment methods, physical treatment such as sonication in this work demonstrates significant advantages. Firstly, it leaves no chemical residues in the water, has a minimal impact on the aquatic environment, and is a more environmentally friendly method for algae control. Secondly, experimental data show that ultrasonic treatment can rapidly reduce algal density and inhibit algal growth, demonstrating its high efficiency. This study demonstrates the significant effectiveness of ultrasonic treatment in algae control and reveals its mechanism and long-term effects. In fact, a short-term ultrasonic radiation has a long-term stress effect on algal cells, and this might be able to prevent the tendency of cyanobacteria blooms.

This finding has important implications for controlling algal blooms and treating water. Ultrasonic radiation is environmentally friendly, efficient, and cost-effective, making it a potential alternative to conventional chemical and biological treatments. The experimental results indicate that *Microcystis aeruginosa*, a dominant species in algal blooms, is sensitive to ultrasonic radiation. The ultrasonic radiation imposes oxidative stress on *Microcystis aeruginosa*, while the impact on other eco-friendly microalgae is relatively minor. This suggests that after ultrasonic treatment, these microalgae beneficial to ecological balance can continue to survive as a food source in the ecosystem, providers of oxygen, and contributors to carbon sequestration in lakes. This provides a theoretical foundation for researchers to further develop ultrasonic technology and apply it to actual water bodies for management. However, our study has limitations. We only investigated the effects of a single frequency and power of ultrasonic radiation, and the results may vary under different conditions. Future research should explore the effects of varying frequencies, powers, and exposure times, as well as the potential for combining ultrasonic treatment with other water treatment methods. Additionally, it would be beneficial to investigate the effects of ultrasonic treatment on algal metabolic products and their potential impact on the environment and water ecosystems.

## 4 Conclusion

This study comprehensively investigated the effects of ultrasonic radiation on mixed algae cultures over a 5-day period, revealing ultrasonic impacts on cell growth, photosynthetic activity, cell membrane integrity, metabolite levels, and genomic stability. Following ultrasonic treatment, there was a significant decrease in the cell count of the algae mixture, with an average reduction of approximately 50 % within the first 24 h. The cell count remained lower than the control group even after 48 h, indicating that while some cells showed signs of recovery, the overall population was still affected by the ultrasonic radiation. The concentration of chlorophyll-a, a key photosynthetic pigment, decreased by approximately 30 % after ultrasonic treatment. Phycobiliproteins, which are involved in light capture and energy transfer in cyanobacteria, also showed a reduction of about 25 %. The results demonstrate that ultrasonic treatment not only causes immediate damage but also has enduring effects on algal cells, with the number of cells significantly reduced following treatment. EEM analysis revealed changes in the fluorescence intensity of both EOM and IOM after ultrasonic treatment. For *Microcystis aeruginosa*, genomic analysis revealed a complex pattern of gene expression changes in response to ultrasonic treatment, with genes involved in stress response, cell repair, and metabolic regulation, indicating the algae's efforts to adapt to the stress. Conversely, genes related to photosynthesis and growth were downregulated, suggesting that these fundamental physiological processes were negatively impacted by the ultrasonic treatment. The understanding of these mechanisms can be further emphasized and deepened to better provide a theoretical basis for future research and applications. The ultrasonic treatment induces physical force, high temperature, and free radical generation, which play a critical role in cellular damage. This oxidative stress disrupts the integrity of the cell membrane and leads to the leakage of cellular contents, ultimately causing cell death. Additionally, the ultrasound-induced changes in gene expression reflect the algae's adaptive response to the stress, with upregulation of stress response and cell repair genes and downregulation of photosynthesis and growth-related genes. These findings enhance our understanding of the complex mechanisms of ultrasonic effects on algae and provide valuable insights for the strategic application of ultrasound in managing algal blooms and improving water quality. The eco-friendly, efficient, and cost-effective nature of ultrasonic radiation suggests that it could be a viable alternative to traditional chemical and biological treatments. Future research should focus on exploring the effects of varying frequencies, powers, and exposure times, as well as the potential for combining ultrasonic treatment with other water treatment methods.

## CRediT authorship contribution statement

**Xiaoge Wu:** Writing – review & editing. **Tingting Shen:** Writing – original draft. **Xiaoyang Liu:** Visualization, Investigation. **Guangming Zhang:** Writing – review & editing. **Xiaoqing Qian:** Visualization, Investigation. **Wenlan Yang:** Writing – review & editing.

## Declaration of competing interest

The authors declare that they have no known competing financial interests or personal relationships that could have appeared to influence the work reported in this paper.
