# Supplementary material for: Unveiling the mechanisms of ultrasonic radiation-induced free radical stress on algal communities: Insights into growth inhibition, photosynthetic disruption, and antioxidant defense responses
Source: Ultrason Sonochem. 2025 Mar 1;115:107297. doi: 10.1016/j.ultsonch.2025.107297 (PMC11924934; doi:10.1016/j.ultsonch.2025.107297)
Supplement: Supplementary Data 1 [file mmc1.docx]

**Supplementary material**

**Unveiling the Mechanisms of Ultrasonic Radiation-Induced Free Radical Stress on Algal Communities: Insights into Growth Inhibition, Photosynthetic Disruption, and Antioxidant Defense Responses**

Xiaoge Wu^ac^, Tingting Shen^b^, Xiaoyang Liu^b^, Xiaoqing Qian^a^, Shiqi Liu^b^, Guangming Zhang^b*^，Wenlan Yang^a*^

^a^ College of Environmental Science and Engineering, Yangzhou University, Yangzhou 225009, China

b School of Energy & Environmental Engineering, Hebei University of Technology, Tianjin 300130, China

^c^ Key Laboratory of Cultivated Land Quality Monitoring and Evaluation, Ministry of Agriculture and Rural Affairs, Yangzhou 225009, China

**Fig. S1.** The yield of H₂O₂ produced by 80 kHz ultrasound within 30 seconds is measured by the KI method.
